# Cumulative Transcutaneous Spinal Stimulation with Locomotor Training Safely Improves Trunk Control in Children with Spinal Cord Injury: Pilot Study

**DOI:** 10.3390/children12070817

**Published:** 2025-06-21

**Authors:** Liubov Amirova, Anastasia Keller, Goutam Singh, Molly King, Parth Parikh, Nicole Stepp, Beatrice Ugiliweneza, Yury Gerasimenko, Andrea L. Behrman

**Affiliations:** 1 Kentucky Spinal Cord Injury Research Center, University of Louisville, Louisville, KY 40202, USA; goutam.singh@louisville.edu (G.S.); molly.king.2@louisville.edu (M.K.); parth.parikh@louisville.edu (P.P.); n.knapp@louisville.edu (N.S.); beatrice.ugiliweneza@louisville.edu (B.U.); andrea.behrman@louisville.edu (A.L.B.); 2 Kosair for Kids Center for Pediatric NeuroRecovery, University of Louisville, Louisville, KY 40202, USA; 3 Department of Neurological Surgery, University of California, San Francisco, CA 94143, USA; anastasia.keller@ucsf.edu; 4 Department of Neurological Surgery, University of Louisville, Louisville, KY 40202, USA; 5 Kosair for Kids School of Physical Therapy, Spalding University, Louisville, KY 40202, USA; 6 Department of Anatomical Sciences and Neurobiology, University of Louisville, Louisville, KY 40202, USA; 7 Pavlov Institute of Physiology, St. Petersburg 199034, Russia; yury.gerasimenko@louisville.edu

**Keywords:** activity-based locomotor training, spinal cord transcutaneous stimulation, spinal cord injury, pediatrics, trunk control

## Abstract

Background/Objectives: Non-invasive spinal cord transcutaneous stimulation (scTS) has expanded the therapeutic landscape of spinal cord injury (SCI) rehabilitation, offering potential benefits beyond compensatory approaches to paralysis. Children with SCI are particularly susceptible to developing neuromuscular scoliosis due to trunk muscle paralysis and ongoing skeletal growth, making targeted interventions crucial. As demonstrated in adults and pediatrics with SCI, the ability of scTS to acutely and safely enable an upright posture and trunk control could be leveraged as a therapeutic adjunct. Activity-based locomotor training (AB-LT) alone significantly improves trunk control in children with SCIs; combining it with scTS may enhance outcomes. This pilot study evaluated the safety, feasibility, and cumulative effects of AB-LT combined with scTS on trunk control in children with SCI. Methods: Three children with SCI completed 19 to 64 sessions of combined AB-LT and scTS. Adverse effects were monitored session to session, and trunk control was assessed pre- and post-intervention. Results: Across 130 interventions in three participants, 88.5% of sessions were free from adverse effects. Reported adverse events included autonomic dysreflexia (5.4%), skin redness at electrode sites (4.6%), and headaches (1.5%). No significant impact of scTS on fatigue or central hemodynamic parameters was observed. Post-intervention, all participants demonstrated improved trunk control during quiet and perturbed sitting. Conclusions: These findings provide the first evidence supporting the safety and feasibility of this combinatorial approach in pediatric SCI rehabilitation while emphasizing the importance of monitoring skin integrity and signs of autonomic dysreflexia. This intervention shows potential synergistic benefits, warranting further research to confirm efficacy and optimize therapeutic protocols.

## 1. Introduction

In both adults and children suffering either cervical or thoracic spinal cord injuries (SCI), the inability to maintain upright sitting posture remains a primary consequence and a function that adults with tetraplegia view as critical to their quality of life [1]. Without recovery of postural control below the lesion, patients are taught compensatory strategies to manage trunk stability and prevent falls, such as weight-bearing through the arms, use of orthoses or straps, or anchoring to the wheelchair. However, these strategies offer limited restorative potential and may offer functional gains via compensation and adaptation to paralysis. For children, the consequences are complicated by the continued growth of an immature musculoskeletal system placing them at high risk for development of scoliosis, e.g., nearly 100% if injured before 10 years old [2,3]. Children who cannot sit up independently or control their trunk upright, even partially, are further challenged to successfully engage with their world via play, self-care, and social interaction.

The recent advancement of spinal cord stimulation from invasive (i.e., epidural stimulation) to transcutaneous (scTS) has opened new therapeutic possibilities for pediatric SCI rehabilitation [4,5]. Although scTS has demonstrated encouraging results in children with acute flaccid myelitis [6] and cerebral palsy [7,8,9], its safety profile requires proof-of-concept and careful evaluation due to the potential for adverse effects. One of the most severe complications in individuals with SCI is autonomic dysreflexia (AD)—a serious hypertensive response, often triggered by bowel or bladder distension, infection, or other noxious stimuli [10]. While scTS may induce AD [11] and other adverse events, the scarcity of data on its safety in pediatric SCI underscores the need for further investigation. Our previous [5,12] work and those of others [6] contribute to addressing this gap by systematically evaluating the safety and feasibility of scTS in pediatric SCI.

Despite these risks, scTS represents a promising adjunct to neuromuscular rehabilitation [13,14,15]. A characteristic effect of scTS is the immediate and reversible trunk extension that ceases upon discontinuation of stimulation [12,16]. The theoretical premise is that spinal stimulation changes the physiological state of the spinal cord to be more responsive to therapeutic interventions [14,15,17]. Training does not use stimulation levels that trigger trunk extension, but instead applies sub-threshold intensity to enhance spinal responsiveness during task-specific training [18]. With studies in adults with SCI [16] and children with SCI [12] demonstrating the immediate effects of spinal stimulation on trunk posture and control, we are primed to study the consecutive and combinatorial effect of stimulation and training.

We chose to combine scTS with activity-based locomotor training [19,20] as the training medium. Our prior work via case studies, consecutively enrolled clinical sample of convenience, and a clinical trial [21,22,23,24,25] demonstrate that activity-based locomotor training (AB-LT) alone improves trunk control in children with SCI with a minimum of 20 sessions and continued improvement through 60 sessions with durability [26]. Daily activity-based locomotor training sessions (mean: 61, range: 48–107) effectively improved trunk control in children with either acute or chronic (>1 year) SCI by a mean of 6/20 points on the Segmental Assessment of Trunk Control (SATCo) [22]. Moreover, an increasing body of evidence, including in pediatric populations, suggests that activity-based training can promote network reorganization below the level of injury, thereby supporting recovery [27]. Combining scTS with AB-LT offers the potential for synergistic effects, enhancing spinal cord excitability and neural remodeling to improve the neuromuscular capacity for trunk control.

In this pilot study, we aimed to achieve two primary objectives: (1) to evaluate the safety, feasibility, and risks associated with the combined application of scTS and AB-LT in children with SCI, and (2) to assess the cumulative effects of 19 to 64 sessions of this combinatorial interventions on trunk control and postural stability. By systematically examining these outcomes, we seek to provide essential insights into the therapeutic potential of scTS in pediatric SCI rehabilitation.

## 2. Materials and Methods

### 2.1. Participants

Three male participants (Table 1) were recruited to this study from a previous study published in Keller et al. [12]. Since one of the objectives of the present study was to assess the feasibility of long-term scTS intervention in a pediatric population with SCI (proof-of-principle), children of different ages were intentionally included. For participation, the inclusion criteria were as follows: (1) history of chronic, acquired upper motor neuron SCI, (2) moderate to severe trunk control deficit as assessed by the SATCo (score < 15/20) [22,28], and (3) completion of ≥60 sessions of AB-LT. Exclusion criteria consisted of: (1) use of botox within the past 3 months, (2) current oral baclofen use, (3) musculoskeletal impairment limiting range of motion, unhealed fracture, or other medical complications limiting participation in the study, (4) prior surgery for scoliosis, (5) congenital SCI, and (6) total ventilator dependence.

### 2.2. Experimental Design

**Figure 1:**
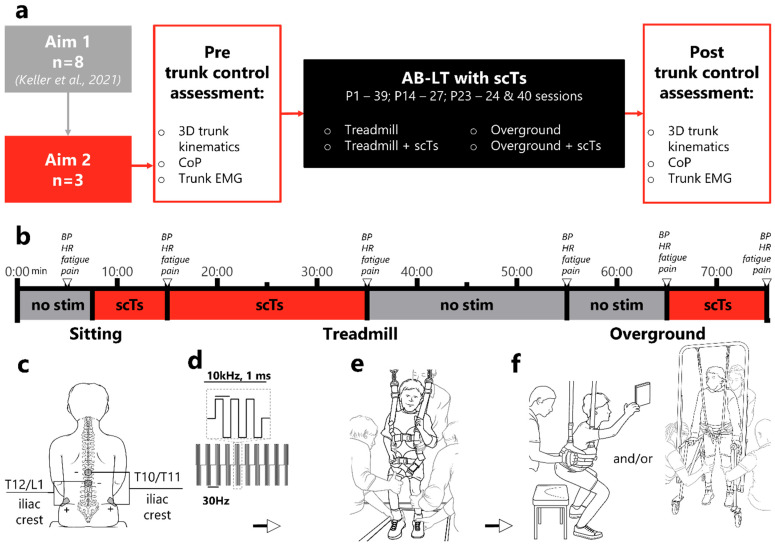
Study design and intervention session. (**a**). Scheme of the experiment. Three of the eight participants from the previous Aim [12] were enrolled in the current study. Participants completed a trunk control assessment pre- and post-AB-LT with scTS intervention. (**b**). AB-LT with scTS intervention. The timeline shows the approximate timing of different locomotor training conditions. After a period of optimization of the stimulation parameters (sitting, scTS), AB-LT on the treadmill (scTS, then with no stimulation) and on the overground (no stimulation, then scTS). BP, HR, fatigue, and pain were measured at the end of each condition (grey arrows). (**c**). Electrode application. Cathodes were placed on the skin between the T10/T11 and T12/L1 vertebrae, and anodes were placed over bilateral ASIS. (**d**). Current modulation. The 10 kHz current was packaged in 15, 30, or 60 Hz packs. (**e**). Treadmill condition. Activity-based Locomotor Training was performed by therapists and activity-based technicians to promote load-bearing, stepping, and standing with partial body weight support, manual facilitation of pelvic rotation, and limb stepping kinematics with upright trunk and coordinated arm swing. Trunk control was also challenged during standing activities with reaching overhead and fine motor tasks [19]. (**f**). Overground condition. Therapists and trainers challenged trunk control during sitting, standing, and stepping overground via activities, e.g., batting balloons, catching and throwing balls, sit-to-stand with the upright trunk. Abbreviations: EMG—Electromyography, AB-LT- activity-based locomotor training, scTS—spinal cord transcutaneous stimulation, BP—blood pressure, HR—heart rate, ASIS—anterior superior iliac spine, ms—milliseconds, Hz—hertz, CoP—center of pressure.

According to the original pilot study design, participants were expected to complete 40 sessions of AB-LT with scTS intervention (Figure 1a). Trunk control assessments were performed in pre-, around post-20, and post-40 intervention sessions. However, due to COVID-19 quarantine guidelines, the intervention was interrupted for P14 (27 sessions) and P23 (24 sessions). P23 returned after a 4-month break and the AB-LT with scTS intervention course began again resulting in two admissions of intervention. As a result of these interruptions, the total number of sessions and the number of sessions completed by the time of trunk assessments may differ across participants.

The comfort and status of the participants were carefully monitored throughout the assessments and AB-LT with scTS intervention sessions. All experiments, training sessions, and events were documented. Any adverse events identified during an intervention/assessment session were followed within 24 h for status updates with the parent/caregiver when indicated (i.e., skin redness under an electrode).

#### 2.2.1. Activity-Based Locomotor Training (AB-LT)

A team of experienced pediatric physical therapists performed AB-LT (Figure 1b). An intervention session lasted 60–75 min and included a 45–60 min session of facilitated standing and stepping on a pediatric treadmill (Pediatric PowerStep, Power NeuroRecovery, USA) with a partial body weight support system and activities off the treadmill challenging trunk control (15–30 min). AB-LT consisted of interspersed bouts of facilitated stepping standing for 5–10 min each. The treadmill speed and amount of body weight support were adjusted based on the participant’s response and the trainers’ capacity to maintain limb kinematics consistent with walking and were recorded for each session (Table 2). Activities challenging trunk control on the treadmill included overhead and side-to-side reaching, shooting basketballs, and fine motor tasks while maintaining trunk control. Overground condition training (off the treadmill) included activities such as assisted standing, sit-to-stand, grasping an overhead object, batting balls with racquets, squatting, and stepping in a walker while maintaining trunk control. Throughout the AB-LT, trainers used play-oriented activities to promote and challenge active trunk extension and rotation via overhead reaching, and controlled hand/arm tasks requiring trunk control for performance (e.g., Jenga) while monitoring for kinematically correct trunk postures. A neutral pelvis position was manually facilitated with an upright trunk applying varying levels of trunk control. Trunk support was provided according to the support identified from the SATCo and ongoing, daily re-assessment of necessary support [22].

#### 2.2.2. Transcutaneous Spinal Cord Stimulation (scTS)

Round electrodes (⌀ 3.2 cm, Axelgaard PALs Platinum, Sacramento, CA, USA) were placed at the midline between T10/11, and T12/L1 vertebrae as cathodes, and two electrodes (5.0 × 9.0 cm, Axelgaard PALs Platinum) were placed symmetrically on the skin over the iliac crests as anodes (Figure 1c). All electrodes were checked for any defects in the insulation layer before placement and were discarded if defects were observed. A transcutaneous stimulator Biostim-5, (Cosyma, Moscow, Russia) was used to deliver biphasic rectangular waveform current with 1-ms pulse with and 15, 30, or 60 Hz frequency with 10 kHz modulated carrier frequency [7,16,29] (Figure 1d). The amplitude of scTS in each spinal level for each participant was selected individually [16]. Initially, a motor threshold of scTS was reached, defined as a visible increase in thoracic and lumbar trunk extension and achievement of upright sitting posture. Further, scTS was performed at the submotor threshold intensity, which was 10–25% below the motor threshold. The mean scTS amplitudes for each participant are summarized in Table 3.

#### 2.2.3. Risk Estimation and Hemodynamic Monitoring

Risk assessment was performed daily during each combined AB-LT with scTS intervention. A list of potential adverse events was based on our previous study [12]. There were two categories of risks anticipated—risks that could be associated with scTS (i.e., skin redness from electrodes, autonomic dysreflexia, spasticity, numb feeling, pain from stimulation, tingling, headache, and bowel accident), and ones that could be associated with AB-LT, (i.e., skin irritation from harness or therapist’s manual contact, muscle soreness, joint sprain, and bone fracture). Before each session, the participant’s skin was examined for any redness, particularly in the areas of electrode placement observed during the prior session. The mean risk in percentage terms for each occurrence was identified based on the data collected.

Blood pressure (BP) and heart rate (HR) (Welch Allyn Connex Spot Monitor) were assessed at (1) arrival in sitting, (2) following scTS optimization in sitting, (3) on the treadmill with scTS, (4) on the treadmill without scTS, (5) during overground activities without scTS, and (6) during overground activities with scTS (Figure 1b). Additional blood pressure measurements were taken if the participant reported or showed any signs of AD (e.g., sudden onset of goosebumps, facial redness). In conjunction with BP measurement, trainers assessed fatigue using the Modified Borg Rate of Perceived Exertion (RPE) scale, and pain using the Visual Analog Scale (>8 years old) or the Wong–Baker Faces Pain Rating Scale (<8 years old).

#### 2.2.4. Trunk Control Assessment

The trunk control stability protocol was adapted from Rath et al., 2018 [16]. Participants were seated during the assessment and the protocol differed according to the participant’s ability to sit independently, without or with support. Participant 1 (P1, hereinafter abbreviated for all participants) and P14 were able to sit independently (without arm support) and for them, the protocol included the following:

- Quiet sitting with arms crossed over the chest for 10 s;
- Leaning forward, backward, right, and left with arms crossed over the chest as far as possible, without falling;
- Raising the right arm rapidly as a self-perturbing task.

P23 had poor trunk control, as indicated by the lowest SATCo, 9/20. He also had impaired arm control limiting his capacity to raise an arm overhead and was not capable of performing complex upper extremity motor and manual tasks. Therefore, the trunk assessment protocol for him included:

- Quiet sitting. The patient’s hands lay on his knees without propping on them and asked to maintain sitting balance as long as he can;
- Sit upright as best as possible, actively attempting to straighten his trunk without support from trainers for 5 s.

Trials were excluded and/or repeated if the participant lost control and had to use their hands on a surface to balance themself at any point during the movement. At least three successful trials for each task were attained and analyzed.

The assessment was performed pre- and post-intervention. During trunk control assessment, 3D trunk kinematics, the Center of Pressure (CoP) in the sitting position, and trunk muscle electromyographic (EMG) activity were simultaneously recorded.

Improved trunk control was inferred if the participant demonstrated an increased duration of independent sitting, reduced trunk sway during quiet sitting and self-perturbations, and greater trunk excursion in response to leaning tasks without loss of balance. Additionally, more optimal trunk control was supported by EMG patterns—specifically, increased engagement of the erector spinae muscles during upright sitting and greater activation of the trapezius muscle during the self-perturbation arm raise task.

#### 2.2.5. 3D Trunk Kinematics

For 3D trunk kinematics, MVN BIOMECH Awinda MTW2-3A7G6 sensors (Xsens Technologies B.V. Enschede, Netherlands) [30,31] were secured using a headband or tape, and on the occipital cranium headbone, at the center of the sternum, scapulas, and at the sacrum-L5 level on the pelvis [32]. Body segment angles were automatically generated inside of Xsens using the Xsens Kalman filter. To assess the effects of AB-LT with scTS on trunk control, angular excursions (difference between maximum and minimum angles during the event) were calculated for the head-T8 (angle at the intersection of the axes of the occipital bone and the 8th thoracic vertebra) and T8-pelvis (angle at the intersection of the axes parallel to the 8th thoracic vertebra and the pelvic acetabulum) angles in the anteroposterior and mediolateral directions.

#### 2.2.6. Center of Pressure (CoP)

Experiments were performed using the FP4060-NC-1000 (Bertec, Columbus, OH, USA) force plate analysis system. CoP data were collected (2000 Hz) using a custom-designed LabView program. The signal was filtered using a 10 Hz low pass, 4th order Butterworth filter. Overall CoP displacement, sway length, and average velocity in anteroposterior and mediolateral, and 95% confidence area of CoP were calculated [33].

#### 2.2.7. Electromyography (EMG)

Trunk muscle activation was recorded using wireless bipolar Pico EMG electrodes (Cometa, Italy, 2000 Hz sampling frequency) that were placed bilateral on the oblique, rectus abdominis, erector spinae (T10 and L5), and upper trapezius muscles. The signal was filtered with a 4th order Butterworth filter, 20 Hz highpass. An overall the root-mean-square (RMS) value per muscle per trial was calculated.

### 2.3. Statistical Analysis

Safety was assessed by examining the risk likelihood of adverse events. The risk likelihood was calculated as the percentage of the number of adverse effects to the total number of sessions conducted, and was classified as “very unlikely to occur” (0–10%), “unlikely to occur” (11–40%), “may occur about half of the time” (41–60%), “likely to occur” (61–90%) and “very likely to occur” (91–100%) [34].

To evaluate the safety and feasibility of the intervention across a session sample, we conducted individual statistical analyses for each participant. Given differences in age, level of injury, and baseline motor function, this approach allowed for a more accurate assessment of participant-specific responses, including potential adverse effects and functional improvements. Systolic blood pressure (SBP), diastolic (DBP), HR, and fatigue were evaluated with mixed linear models including a random intercept per session and including a condition variable as the independent variable. The condition variable had the following categories: (1) sitting without scTS, (2) sitting with scTS, (3) treadmill with scTS, (4) treadmill without scTS, (5) overground without scTS, and (6) overground with scTS. Data were presented with the least square means with associated standard error. The main intent was to evaluate the estimated average value within each non-stimulated timepoint compared to scTS, and sitting position compared to treadmill and overground conditions. Additionally, the correlation between fatigue and stimulation intensity was calculated with repeated measure correlation which adjusts for multiple measures on stimulation channels (channels 1 and 2) using the rmcorr package [35], and correlation strength (strong, weak) identified according to J.D. Evans [36].

The effect of AB-LT with scTS interventions on trunk control was evaluated in an exploratory fashion. For this, trunk control measures (head-T8 and T8-pelvis segments excursion, CoP, EMG) were evaluated with mixed linear models including study timepoint (pre- and post-intervention) as a fixed factor and trial (3–4 trials each time) nested within the study timepoint as a random factor. Estimates were presented with least square means with associated standard errors.

All tests were 2-sided with the significance level of α = 0.05. Statistical analyses were performed in SAS 9.4 (SAS Inc, Cary, NC, USA) and R Statistical Software (version 4.3.1, R Core Team 2023).

## 3. Results

Safety & feasibility of combined scTS with AB-LT interventions

### 3.1. Adverse Effects

Three male children underwent a total of 130 AB-LT with scTS sessions. In 88.5% (115 sessions), the sessions were safe and had no scTS-associated adverse effects. We did not observed pain from stimulation, numbness, spasticity, or bowel accident. The adverse effects we monitored are summarized in Table 4. Further, we will describe in more detail the most adverse events that we encountered in this work.

We registered 7 episodes of AD in two participants with the third participant having no episodes. P1 experienced two episodes of AD in different sessions. In one session, he had a sudden urge to urinate during a treadmill session without scTS accompanied by a BP of 157/90 mmHg and a heart rate of 76 bpm. After urination, P1’s BP decreased to 109/40 mmHg with an HR of 78 bpm. A second episode of AD in another session was recorded at the end of the session (i.e., the training and stimulation had been completed by this point) before discharge. On this occasion, P1’s BP was 146/78 mmHg with an HR of 59 bpm, representing an increase of 38/25 mmHg from the previous measurement. After 6 min of rest, BP decreased and he was cleared for discharge from the session. P23 experienced 5 episodes of AD, all occurring in different sessions within 10. During these episodes, P23 experienced redness of cheeks and ears. Monitoring his hemodynamics parameters showed an increase in BP of 47/29 mmHg and a decrease in HR by 20 bpm on average for those 5 sessions (except for one session when HR was increased a 10 bpm). The participant was immediately placed in a sitting position, and both stimulation and exercise were discontinued until central BP and HR normalized. Four of the five AD episodes were recorded in the absence of scTS.

Skin redness under the stimulating electrodes was the second most common observation (4.6%), with 5 out of 6 incidents occurring in P14. This redness was never associated with pain (though P14 once reported a tingling sensation) dissipating within 24 h of re-check of skin status.

### 3.2. Fatigue & Pain Levels

**Figure 2:**
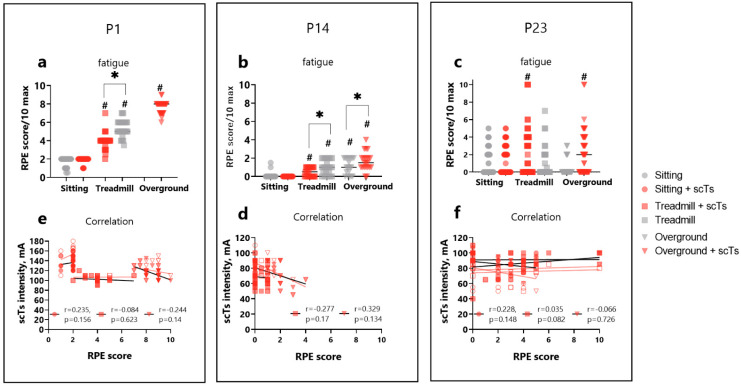
Fatigue levels and correlation scTS intensity. (**a**–**c**). Fatigue levels averaged across all AB-LT sessions for each participant, as measured by the Modified Borg Rate of Perceived Exertion scale. Data points represent individual fatigue scores at each training time point: sitting and sitting + scTS (circles), treadmill and treadmill + scTS (squares), overground and overground + scTS (triangles). Gray marker indicates the no-stimulated condition, while red markers indicate scTS condition; (**d**–**f**). Correlation between fatigue scores and the scTS intensity of each channel. Red filled dots (circles, squares, and triangles) and black trend line—stimulation intensity for T10–T11 stimulation site; red unfilled dots and red trend line—stimulation intensity for T12-L1; circles—sitting, squares—treadmill, triangles—overground. Statistical analysis: mixed linear model including a random intercept for each training point: sitting, sitting + scTS, treadmill + scTS, treadmill, overground, overground +scTS. The correlation between fatigue and mean scTS intensity was used with a mixed linear model #—vs. sitting, *—vs. scTS condition, *p* < 0.05. Correlation between fatigue and stimulation intensity was calculated with repeated measure correlation which adjusts for multiple measures on stimulation channels (channels 1 and 2) using the rmcorr package [35]. Abbreviations: scTS—spinal cord transcutaneous stimulation, mA-milliampere.

Fatigue and pain levels were assessed throughout the AB-LT with scTs intervention before each change in condition. Fatigue levels varied among participants: P1 reported a progressive increase in fatigue level (Figure 2a) throughout the training session (first vs. last condition, *p* < 0.0001, Cohen’s d = 18.6). Typically, P1 had a fatigue level of 0.5–2 immediately after arriving. Significant differences in fatigue were observed between with and without scTS treadmill conditions (5 points vs. 4 points, *p* < 0. 0001, d = 4.4). However, weak and very weak correlations were found between the scTS intensity and fatigue score, respectively (R = 0.235; −0.084; −0.244, sitting, treadmill, and overground, respectively) (Figure 2e). P14 also showed a trend of progressively increasing fatigue during AB-LT training (first vs. last condition, *p* < 0.0001, d = 2.93) (Figure 2b). Significant differences in fatigue scores were found between with and without stimulation in both treadmill and overground conditions (*p* < 0.0001, d = 1.15 and d = 0.88). Weak correlations (R = 0.277; 0.329 treadmill and overground, respectively) were observed between scTS intensity and fatigue score for this participant (Figure 2d). P23 showed no consistent pattern between fatigue score and training time (Figure 2c). He rated his fatigue level as “0” in 59.4% of cases, with varying severity from 0.5 to 10 in the remaining instances. Weak and very weak correlations were found (R = 0.228; 0.035; −0.066, sitting, treadmill, and overground, respectively) between scTS intensity and fatigue score, respectively (Figure 2f).

P14 reported minor headaches during two interventions. One headache was noted immediately upon arrival (1 point) and continued during treadmill training (scTS was not applied until the pain subsided). During subsequent overground conditions, both with and without scTS, the participant did not experience pain. A second headache occurred during overground training with no stimulation (1 point). Other than the above, participants generally reported no pain during the interventions.

### 3.3. Blood Pressure and Heart Rate

**Figure 3:**
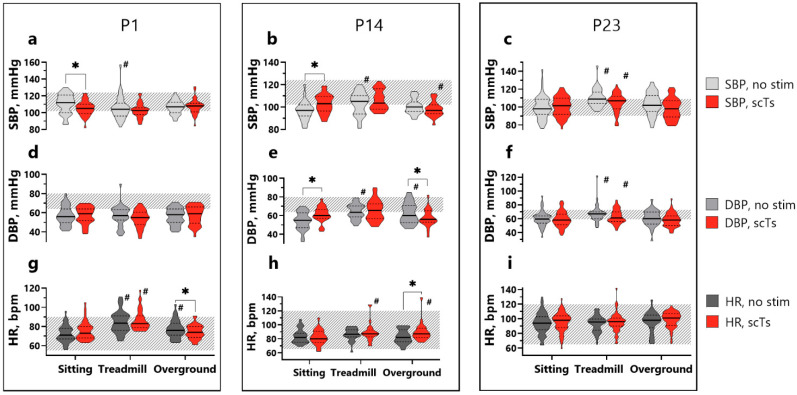
Central hemodynamic parameters averaged over all AB-LT. (**a**–**c**). SBP for each time point training for P1, P14, and P23; (**d**–**f**). DBP; (**g**–**i**). HR. Data are represented as violin plots with median (bold line) and quartiles (dashed line). Gray shades—no-stimulated condition, red color—scTS. Hatching plane—50th to 90th sex- and age-matched normal range for typical developing children. Statistical analysis: mixed linear model including a random intercept for each training point: sitting, sitting + scTS, treadmill + scTS, treadmill, overground, overground +scTS. #—vs. sitting, *—vs. scTS condition, *p* < 0.05. Abbreviations: SBP—systolic blood pressure, DBP—diastolic blood pressure, HR—heart rate, AB-LT—activity-based locomotor training, scTS—spinal cord transcutaneous stimulation.

In P1, SBP during AB-LT was mostly within the 50–90th percentile range for typically developing children (as indicated by the hatching plane in Figure 3), or lower (Figure 3a). In the sitting position without scTS, the medial SBP was 112 mmHg, which significantly decreased with scTS (*p* = 0.0192, d = 0.89). SBP also significantly decreased during treadmill condition without scTS compared to sitting (*p* = 0.0316, d = 0.82). DBP was below the normal range in the sitting position (56 mmHg without scTS and 59 mmHg with scTS) and showed no significant changes during training or scTS (Figure 3d). HR in the sitting position was within the normal range (71 bpm without scTS and 73 bpm with scTS) and significantly increased during both stimulated and non-stimulated treadmill conditions (*p* < 0.0001, d = 3.9 and d = 2.0) and non-stimulated overground (*p* = 0.0035, d = 1.1) (Figure 3g). Additionally, HR was significantly lower during scTS overground compared to the non-stimulated overground condition (*p* = 0.0392, d = 0.78).

In P14, SBP (Figure 3b) was predominantly within the sex- and age-matched normative range. Stimulation in the sitting position increased SBP by 6 mmHg (*p* = 0.0092, d = 1). During non-stimulated treadmill and stimulated overground conditions, SBP was significantly different compared to the sitting position (*p* = 0.0042, d = 1.1 and *p* = 0.0464, d = 0.76). DBP (Figure 3f) was 55 mmHg in the sitting position, which is lower than the normal range, but it reached normal values during scTS (*p* = 0.0058, d = 1.06). DBP during non-stimulated treadmill and overground conditions was increased (*p* < 0.0001, d = 1.52 and *p* = 0.0009, d = 1.28, respectively). Sitting HR (Figure 3h) was within the normal range (82 bpm without scTS and 80 bpm with scTS) and was significantly increased during the stimulated treadmill and overground conditions (*p* = 0.007, d = 1.03 and *p* = 0.0096, d = 0.99, respectively).

In P23, sitting SBP (Figure 3c) was within the age- and sex-specific normal range, measuring 98 mmHg without scTS and 101.5 mmHg with scTS. SBP increased significantly during both the stimulated and non-stimulated treadmill conditions (*p* = 0.0178, d = 0.9 and *p* < 0.0001, d = 1.7 respectively). The sitting DBP was 59.5 mmHg without scTS and 58 mmHg with scTS, within the range of typically developing children (Figure 3f). There were also significant changes in DBP during treadmill both stimulated and non-stimulated conditions (*p* = 0.0129, d = 94 and *p* = 0.001, d = 1.25). HR (Figure 3i) was also within the normal range 94 bpm without scTS and 98 bpm with scTS in the sitting position and remained stable through the AB-LT training.

### 3.4. Trunk Control

With the three participants varying in their capacity for trunk control, i.e., able to sit independently without falling with hands in the air (P1 and P14) to unable to sit independently (P23), different protocols to assess trunk control were used to increase the sensitivity. P1 (completed 39 interventions) and P14 (completed 19 interventions) performed leaning tasks in the anterior (forward lean), posterior (backward lean), left, and right directions, as well as a self-perturbation task involving a quick arm raise, while trunk segment 3-D kinematics, CoP and trunk EMG were recorded.

**Figure 4:**
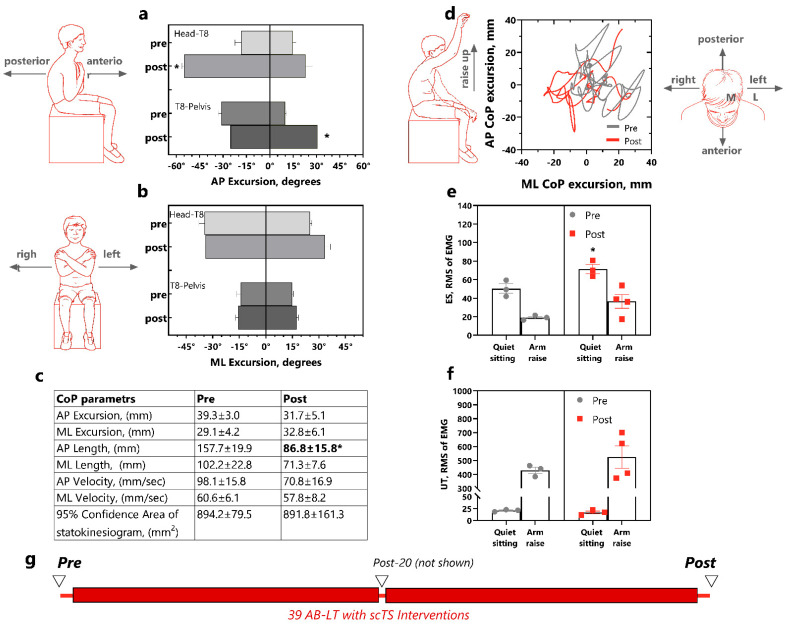
Trunk control in P1 pre- and post-39 AB-LT with scTS interventions. (**a**). Trunk angular excursion during anterior and posterior leaning task for head-T8 and T8-pelvis segments for three trials; (**b**). Trunk angular excursion during left and right leaning task for head-T8 and T8-pelvis segments; (**c**). Pre-post CoP parameters during “right arm up” self-perturbating task; (**d**). CoP trace for “right arm up” self-perturbating task over three trials; (**e**). RMS of EMG of rector spinae at T10 and (**f**). RMS of EMG of upper trapezius. (**g**). Study timeline showing AB-LT with scTS interventions (red rectangles) and trunk control testing (triangles). Data are represented as mean for three-four trials with SEM. Statistics: mixed linear models were used with timepoint (pre-, post-intervention) as a fixed factor and trial nested in timepoint as a random factor (three trials). *—vs. pre-intervention, *p* < 0.05. Abbreviations: AB-LT—activity-based locomotor training, AP—anteroposterior, ML—mediolateral, CoP—Center of Pressure, EMG—electromyogram, RMS—root-mean-square envelope of the EMG, T10—tenth thoracic vertebrata, ES—erector spinae, UT—upper trapezius.

For Participant 1, the trunk kinematics analysis of the anteroposterior leaning direction showed similar excursion of the head-T8 segment during the pre-intervention assessment (anterior 14.9° ± 2.2° and posterior 18.3° ± 4.0°, Figure 4a). After the 39 AB-LT with scTS-intervention sessions, the excursion of this segment increased, with a significant difference in the posterior lean direction (54.8° ± 1.6°, *p* = 0.001, d = 3.47). Prior to the intervention, P1 exhibited substantial asymmetry in the T8-pelvis excursion during anteroposterior leaning, with greater posterior excursion (31.2° ± 1.8°) compared to anterior excursion (9.8° ± 0.8°). This asymmetry diminished post-intervention (posterior 25.2° ± 7.4° and anterior 30.7° ± 1.2°), and the anterior leaning increased significantly compare to the pre-intervention values (*p* = 0.0001, d = 5.93).

The mediolateral excursion of the head-T8 segment was 25.1° ± 0.9° for the right lean and 34.7° ± 3.0° for the left lean leaning during the pre-intervention assessment (Figure 4b). After the intervention, P1’s ability to lean to the right increased by 8°, while the left leaning remained unchanged. The T8-pelvis excursion was consistently smaller than the more rostral segment, measuring 14.8° ± 0.8° for the right lean and 14.3° ± 2.2° for the left lean in the pre-intervention assessment. Post-intervention, the T8-pelvis excursion increased slightly by 2.6° for the right lean and 1.4° on the left lean.

In the self-perturbation test with a right arm rise-up, performed during both pre- and post-intervention, the 95% confidence area of the CoP sway in P1 remained almost unchanged (pre—894.2 ± 79.5 mm^2^ and post—891.8 ± 161.3 mm^2^, Figure 4d). However, one of the CoP characteristics presented in Figure 4c, the length of the anteroposterior CoP displasement, decreased significantly (*p* = 0.0283, d = 1.25).

The RMS envelope of EMG of erector spinae muscles was slightly higher during quiet sitting but decreased during arm raising tasks (Figure 4e). After the AB-LT intervention, this ratio was maintained, with a significant increase in muscles activity during quiet sitting (*p* = 0.0415, d = 1.21).

In contrast, the upper trapezius muscle RMS of EMG had an inverse relationship- the activity was low during quiet sitting and increased with arm raise (Figure 4f). Post-intervention, the EMG activity of the upper trapezius was even lower during quiet sitting. No clear patterns were found among other muscles. The results for this participant are presented in the Supplementary Table S1.

**Figure 5:**
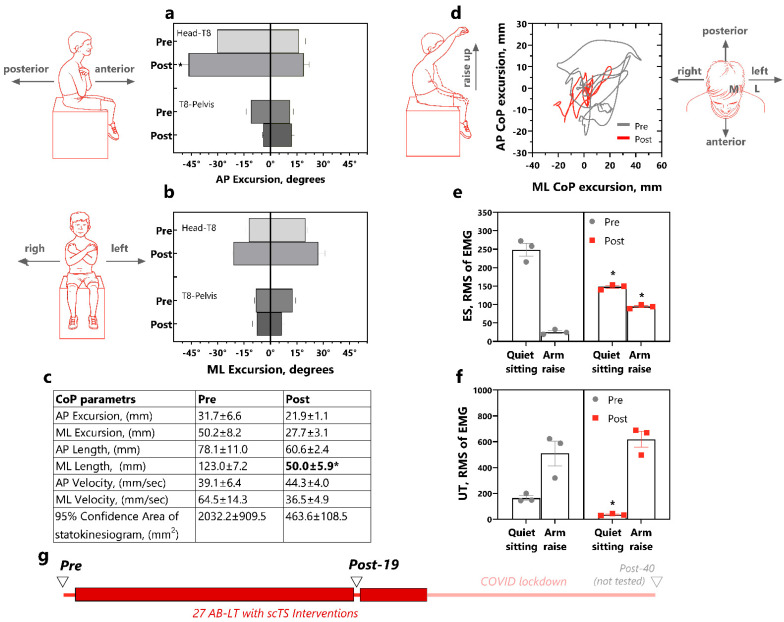
Trunk control in P14 pre- and post-19 AB-LT with scTS interventions. (**a**). Trunk angular excursion during anterior and posterior leaning task for head-T8 and T8-pelvis segments for three trials; (**b**). Trunk angular excursion during left and right leaning task for head-T8 and T8-pelvis segments; (**c**). Pre-post CoP parameters during “right arm up” self-perturbating task; (**d**). CoP trace for “right arm up” self-perturbating task over three trials; (**e**). RMS of EMG of rector spinae at T10 and (**f**). RMS of EMG of upper trapezius. (**g**). Study timeline showing AB-LT with scTS interventions (red rectangles) and trunk control testing (triangles). Data are represented as mean for three trials with SEM. Statistics: mixed linear models were used with timepoint (pre-, post-intervention) as a fixed factor and trial nested in timepoint as a random factor (three trials). *—vs. pre-intervention, *p* < 0.05. Abbreviations: AB-LT—activity-based locomotor training, AP—anteroposterior, ML—mediolateral, CoP—Center of Pressure, EMG—electromyogram, RMS—root-mean-square envelope of the EMG, T10—tenth thoracic vertebrata, ES—erector spinae, UT—upper trapezius.

The 3-D kinematics analysis of Participant 14 revealed a consistently larger excursion for the head-T8 segment compared to the T8-pelvis segment. During the pre-intervention assessment, the anterior leaning excursion was 16.2° ± 3.8° for head-T8 and 11.1° ± 1.9° for T8-pelvis (Figure 5a). Interestingly, the excursion of both segments tended to increase after the 19 AB-LT with scTS interventions. The posterior leaning excursion for head-T8 increased significantly in the post-intervention assessment (*p* = 0.0192, d = 1.55) and was slightly greater than the anterior leaning excursion.

A slight asymmetry was noted in lateral leanings during the pre-intervention assessment—P14 made a larger lean to the left than to the right (20.0° ± 0.8° vs. 12.4° ± 1.6 for head-T8; 12.6° ± 1.8° vs. 8.4° ± 0.5° for T8-pelvis) (Figure 5b). However, the mediolateral excursion in head-T8 increased significantly (*p* = 0.0082, d = 1.99 for right lean), while T8-pelvis decreased slightly, resulting in more symmetrical leans in the post-intervention assessment.

The 95% confidence area of the CoP sway became smaller in the post-intervention assessment during the “right arm up” self-perturbation task (Figure 5d). Comparative pre-post data of the most commonly used CoP parameters showed a significant decrease in the mediolateral CoP dispasment (*p* = 0.0014, d = 3.22) (Figure 5c).

The RMS of EMG of the erector spinae muscles was higher during quiet sitting compared to arm raising (Figure 5e). Interestingly, in post-intervention assessment, the erector spinae EMG activity during quiet sitting significantly decreased (*p* = 0.0045, d = 2.35), but it significantly increased during arm raising (*p* = 0.0002, d = 5.62).

The upper trapezius muscles RMS of EMG value had an inverse relationship, with low activity during quiet sitting but an increase during arm raise (Figure 5f). After the intervention, the upper trapezius EMG activity decreased in quiet sitting, (*p* = 0.0023, d = 2.83) but increased with arm raising. No clear patterns were found among the other muscles. The results for this participant are presented in Table S2.

Participant 23 had the most severe impairment in trunk control and the assessment was limited to the attempts of independent quiet and upright sitting, while trunk segment excursions and trunk EMG were recorded. Also, his research protocol was complex because his first AB-LT with scTS training was interrupted after 24 interventions due to the pandemic COVID-19 and restarted 4 months later with 40 interventions. However, to show his progress we show all the data collected.

**Figure 6:**
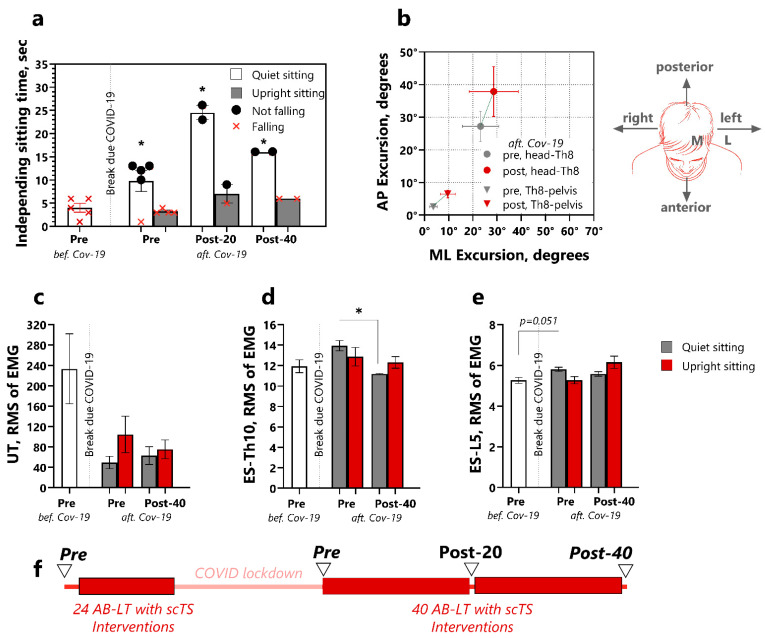
Trunk control in P23 before and after AB-LT with scTS sessions. (**a**). Independent sitting time (unaided) in a relaxed posture and while attempting to sit upright. Red crosses indicate attempts in which P23 lost balance, and black circles indicate the participant successfully maintained balance and completed the trial; (**b**). Anteroposterior and mediolateral excursion of head-T8 and T8-pelvis during an attempt to sit upright; (**c**). RMS of EMG of the upper trapezius; (**d**). erector spinae at Th-10; (**e**). and erector spinae at L-5 while quiet and upright sitting; (**f**). Study timeline showing AB-LT with scTS interventions (red rectangles) and trunk control testing (triangles). Data are represented as mean for three trials with SEM. Statistics: mixed linear models were used with timepoint (pre-, post-intervention) as a fixed factor and trial nested in timepoint as a random factor (three trials). *—vs. pre-intervention, *p* < 0.05. Abbreviations: BB—before break due to COVID-19, AB—after break due to COVID-19, AP—anteroposterior, ML—mediolateral, EMG—electromyogram, RMS—root-mean-square envelope of the EMG, UT—upper trapezius, ES—Erector spinae, T10—tenth thoracic vertebrata, L5—fifth lumbar vertebrata.

The mean initial time of independent quiet sitting for P23 was 4.0 ± 0.95 s prior to loss of balance (Figure 6a). Attempts to sit upright resulted in loss of balance and falling. After the post-COVID interruption, the pre-intervention assessment was repeated and revealed a sustained improvement in P23’s ability to sit independently for approximately 10 s (*p* = 0.0321, d = 0.58). He even could initiate an attempt to sit upright (straighten out the spine) for an average of 3.3 s before losing balance. During the 20–40 interventions of the second admission, P23 further improved his independent sitting time to 20 s (*p* = 0.0001, d = 1.55) and was able to sit upright independently without loss of balance.

The analysis of the excursion of the head-T8 and T8-pelvis segments during the upright sitting attempts in the anteroposterior and mediolateral directions was not performed during the first admission, as the participant lost balance. With the second pre-intervention assessment, the lean excursion was 23.2° ± 7.5°/27.2° ± 4.6° (anteroposterior and mediolateral, respectively) for the head-T8 segment, and 3.6° ± 1.6°/2.5° ± 0.6° for the T8-pelvis (Figure 6b). Following the AB-LT with scTS intervention, the excursion of both segments increased by 5–10 degrees in both the anteroposterior and mediolateral directions.

The analysis of the upper trapezius muscle RMS of EMG value showed substantial activity of 232.2 ± 68.5 mV (Figure 6c) during the first assessment. At the second assessment, the EMG of this muscle decreased markedly. After the 40 interventions, the upper trapezius EMG activity decreased further to 63.1 ± 17.4 mV during quiet sitting and 66.1 ± 27.8 mV during upright sitting.

In contrast to the upper trapezius, the erector spinae muscle showed a different pattern. At the first admission, the EMG activity of the T10 erector spinae muscle was 12 ± 0.6 mV (Figure 6d). After the break, there was a tendency to activate the erector spinae muscle when trying to sit upright, observed in the post-20 s admission (but not post-40) AB-LT with scTS intervention assessments. A similar pattern was observed in the L5 erector spinae muscle, but its activity was lower (Figure 6e). No distinct patterns were found among the other muscles assessed. The detailed results for this participant are presented in Table S3.

## 4. Discussion

The combinatorial approach using scTS with AB-LT across cumulative training sessions (totaling 130 sessions) resulted in 88.5% of the interventions without adverse events. The likelihood of adverse events including AD, skin redness from electrodes, and headache was “very unlikely” as the percentage is <5.38%. Transcutaneous spinal cord stimulation did not negatively impact blood pressure and heart rate during a prolonged course of daily AB-LT. During the intervention, the AB-LT component had a more pronounced impact on participants’ fatigue level than the transcutaneous spinal cord stimulation component. Additionally, AB-LT with scTS intervention improved trunk control in children with SCI during quiet sitting and self-perturbation testing.

Although the vast majority of the sessions were without adverse events, analyzing the cases where they occurred may provide additional information. Adverse effects varied not only from participant to participant but also may not be clustered evenly in the same participant. For example, all 5 episodes of ADs in P23 occurred consecutively over ten sessions out of 64. Another participant (P14) had the most frequent adverse effect of scTS was reddening of the skin at the electrode sites, while in other participants this reaction did not occur, or was noted very rarely.

Since we anticipated the risk of AD [37,38], constant monitoring of BP and HR throughout AB-LT was a priority. We carefully monitored the participants for any typical signs of AD (sudden onset of facial flushing, headache, etc.) [39]. All participants were at risk for AD, however, the circumstances of AD differed.

AD episodes occurred in two participants. P1 experienced two episodes of AD, one of which occurred without stimulation and was likely triggered by a full bladder, a common cause of AD in both adults and children with SCI [39]. The second episode occurred towards the end of the training session with stimulation at T11 and L1 sites during overground activities. In both cases, AD was mitigated within 10 min. All episodes of AD in P23 occurred against a background of urinary tract infection. Although the participant was treated and monitored by a physician, urinary tract infection could both be a trigger for fluctuation in BP and lower the threshold for other triggers. Autonomic dysreflexia is typically triggered by bladder distention or bower impaction, which correlates with our observations and has been reported by some studies to occur in 51% of children with SCI [39]. Both noxious and innocuous sensory input arising below the level of injury can trigger AD following SCI [40,41,42]. Recent studies in adults with SCI demonstrate the safety and applicability of scTS for the cardiovascular system [43,44]. However, some evidence suggests that spinal cord stimulation may not improve autonomic regulation after SCI, and instead might induce tonic sympathetic excitation, potentially lowering the threshold for AD [45]. Therefore, it is advised that a child’s hemodynamic parameters need to be closely monitored throughout any intervention to ensure their safety.

Except for AD cases, the BP and HR of the participants conformed to the sex-age range [46]. The response of cardiovascular parameters to exercise in the older participant P1 differed from P14 and P23. P1 had a significant increase in HR during the stepping bouts on the treadmill either with or without stimulation. The HR increase is a typical physiological response for an exercising individual [47], and therapists and AB-LT technicians always encourage participants’ active effort and intent to step during facilitated locomotor training. An increase in HR could have also been an autonomic response to an orthostatic challenge to maintain BP while upright [48]. Despite the increase in HR, P1’s BP remained unchanged. In contrast, P14 and P23 had an increase in systolic and DBP, while HR remained stable. Several reasons may account for these differences, such as age, height, and time since injury. Higher, cervical levels of injury in P14 and P23 participants were associated with poorer cardiovascular regulation [49,50,51]. In general, we did not observe significant hypotension in our participants, which is characteristic of adult patients with SCI. We may note a slightly reduced diastolic component of pressure in P1 and a mildly reduced baseline BP in P14. Recent studies show that, on the contrary, the application of spinal cord stimulation modestly increases and stabilizes BP [52,53] and HR [54] in individuals with chronic SCI, which in our case could account for some pressure rise in P14 and P23 during AB-LT with scTS.

We monitored skin condition under the stimulation electrodes by carefully examining and noting any changes in skin appearance from the participants’ arrival to the end of a session. Besides temporary skin redness which was not associated with any pain and quickly dissipated, we did not observe any skin damage. A small skin lesion reported in previous studies using scTS in adults with SCI was due to the defect in the electrode [55]. Given this prior observation, we carefully examined the adhesive layer of the electrodes to ensure its integrity, a reasonable preventative measure that in our study effectively curbed any serious adverse skin issues in response to stimulation. Therefore, we emphasize quality control of stimulation electrodes as especially important in studies with individuals who experience sensory deficits due to SCI or other reasons.

Participants rated their level of fatigue throughout the session. Older participants reported increasing fatigue as the therapy session progressed, as expected. However, fatigue was likely related to session time than to scTS intensity directly. Spinal cord stimulation was integrated throughout the AB-LT, and we cannot conclude if scTS increased the participants’ exertion amount. Regardless, consistent experience of fatigue during intensive AB-LT with scTS did not deter children from participating in the study.

In our study, one participant (P14) experienced a headache during the two sessions of training. However, in one case he experienced a headache immediately after arrival (before all intervention activities) and then denied the presence of an ache during the training. P14’s headache was likely an independent event not caused by the intervention.

Our exploratory analysis of trunk control outcomes for the three participants who received AB-LT with scTS suggest that as little as 20–40 continuous sessions of AB-LT with scTS improved neuromuscular capacity for trunk control. P1 and P14 increased their excursion for trunk leaning in the anteroposterior direction. Prior to receiving the AB-LT with scTS, both participants exhibited greater activity in the upper trapezius muscle during quiet sitting, particularly in P14. This suggests that in the absence of adequate trunk control/posture, these individuals compensate by activating non-typical muscles above the level of their spinal cord lesion in order to make up for the paralysis of the trunk musculature [56]. After the intervention, participants exhibited a reduction in upper trapezius muscle activity. Additionally, the muscle activation pattern shifted to a more beneficial state—at rest, the activity was appropriately reduced, while muscle activation increased during attempts at movement. Increased activation of the erector spinae muscle combined with increased amplitude of voluntary leans may indicate improved trunk control. Interestingly, though P14 received half as many AB-LT sessions as P1, his trunk control showed greater improvement trends. P23, who had the most severe deficits in postural balance at baseline increased his capacity for independent sitting time and, likely due to greater activation in the paraspinal muscles, was able to increase the amplitude of trunk sway without loss of balance.

This study is among the first to apply scTS in children with SCI, and its clinical use has outpaced our full scientific understanding of its mechanisms. The effectiveness of scTS in motor rehabilitation appears to be based on multiple factors, including its ability to activate inhibitory networks—specifically, by modulating reciprocal and presynaptic inhibition of spinal α-motor neurons [57,58]. This enhanced inhibition may render spinal circuits more responsive to residual descending inputs and incoming afferent signals [59]. Spinal networks responsible for trunk control may similarly be retrained, as are those governing locomotion, which may explain the observed improvements in voluntary trunk control. A broader mechanism of scTS action may involve activation of various bioactive molecules and growth factors that facilitate plasticity [59]. In our study, scTS intensity remained consistent across sessions, which we hypothesize ensured stable afferent input necessary for network remodeling via the mechanisms described above. However, we intentionally used submotor-threshold amplitudes to preserve intrinsic motor drive, as high stimulation intensities can overwhelm endogenous signals [18].

The limitations of this study include a small sample size of three participants and the absence of a control group, both of which inevitably narrow the generalizability of our observations. Nevertheless, these findings represent an important step in establishing proof-of-concept for the application of scTS in pediatric populations. Another limitation was the temporal variability in the assessment of trunk control, which was influenced by the COVID-19 pandemic. It is also important to note that, as our primary objective was to assess feasibility, we initially planned only 40 AB-LT sessions with scTS, whereas more than 80 sessions are typically recommended to achieve a restorative effect. Despite the limited number of sessions, participants demonstrated measurable improvements in trunk control.

## 5. Conclusions

Our pilot study demonstrates that the combined use of scTS and AB-LT is both safe and feasible for children with chronic SCI and impaired trunk control following cervical and thoracic injuries. Across 130 intervention sessions involving three participants, 88.5% were free from adverse effects. The remaining sessions presented a low incidence of complications, including autonomic dysreflexia (related and unrelated), skin redness, headache, and tingling sensations, with rates ranging from 1.5% to 5%. Future study of scTS should continue to monitor for autonomic dysreflexia and skin integrity due to the high impact of its occurrence on health. Notably, participants who underwent 19 to 64 sessions of the combined therapy exhibited improved upright sitting, greater trunk excursion, and enhanced trunk muscle activation both above and below the spinal cord lesion level, reflecting improved trunk control. These findings provide novel evidence supporting the therapeutic potential of scTS when integrated with locomotor training, specifically within the pediatric SCI population.

## Figures and Tables

**Table 1:** Participant demographics.

Publication ID (Sex)	Age, yrs	Height, cm	Weight, kg	Time Since SCI, yrs	SATCo	SCI Level (ASIA Impairment Scale)	SCI Etiology
P1 (m)	14	164	53	9	12/20	C3–T5 (B)	epidural hematoma
P14 (m)	10	122	19	6	11/20	C7 (A)	motor vehicle accident
P23 (m)	6	109	18	6	9/20	C4–C7 (B)	transverse myelitis

Abbreviations: ID—Publication identification number of participants as assigned in the Research Participant Database, m—male, SCI—spinal cord injury, ASIA—American Spinal Injury Association, SATCo—Segmental Assessment of Trunk Control. Data is reported as individual.

**Table 2:** Activity-based locomotor training parameters.

Publication ID (*n* of AB-LT)	Treadmill Speed, mph	Treadmill Body Weight Support, %	Treadmill Time, min	Overground Time, min	Total AB-LT Time, min
P1 (39)	2 ± 0.01	78.6 ± 1.3	44.3 ± 1.7	16.5 ± 1.1	64.8
P14 (27)	1.1 ± 0.19	49.8 ± 2.5	60.9 ± 1.3	12.3 ± 1.0	78.1
P23 (64) *	1.8 ± 0.06	55.7 ± 1.4	46.1 ± 1.6	21. ± 1.8	74.2

*—For P23, the first 24 AB-LT were conducted before the COVID-19 and then there was a break due to the lockdown. After the break, the intervention was restarted and 40 AB-LT were conducted. Abbreviations: ID—Identification, AB-LT—activity-based locomotor training, *n*—number, SD—standard deviation, mph—miles per hour, min—minutes. Data is reported as Mean ± SEM over training sessions for each participant.

**Table 3:** The amplitude and time of scTS during AB-LT.

	Motor Threshold in Sitting, mA		Treadmill Submotor Intensity, mA		Overground Submotor Intensity, mA		Average scTS Duration per One AB-LT, min	Total scTS Duration per All AB-LTs, Hours
ID (*n* of scTS)	T10/T11	T12/L1	T10/T11	T12/L1	T10/T11	T12/L1	T10/11 & T12/L1	T10/11 & T12/L1
P1 (39)	137.1 ± 1.7	152.8 ± 1.8	101.4 ± 0.7	105.1 ± 0.7	107.5 ± 1.2	111.6 ± 1.1	44.1 ± 0.9	28.7
P14 (27)	78.3 ± 2.0	82.1 ± 2.5	68.3 ± 2.6	70.6 ± 2.6	71.9 ± 2.9	72.2 ± 3.2	45.2 ± 1.5	17.3
P23 (64)	92.8 ± 2.4	91.1 ± 3.4	88.2 ± 1.4	83.2 ± 2.2	91.5± 2.2	88.8 ± 3.5	34.8 ± 1.5	36.5

Data is reported as Mean ± SEM over training sessions for each participant. Abbreviations: scTS—spinal cord transcutaneous stimulation, T—thoracic, L—lumbar, SEM—standard error of the mean.

**Table 4:** Occurrence of risks associated with scTS and AB-LT.

		P1’s Events	P14’s Events	P23’s Events			
	Adverse Occurrence	(*n* = 39 Sessions)	(*n* = 27 Sessions)	(*n* = 64 Sessions)	Occurrence Rate	Risk	Likelihood
Risks associated with scTS	Autonomic dysreflexia	2	0	5	7/130	5.38%	Very unlikely to occur
	Skin redness from electrodes	0	5	1	6/130	4.61%	Very unlikely to occur
	Headache	0	2	0	2/130	1.54%	Very unlikely to occur
	Spasticity	0	0	0	0/130	0%	Very unlikely to occur
	Pain	0	0	0	0/130	0%	Very unlikely to occur
	Numbness from stimulation	0	0	0	0/130	0%	Very unlikely to occur
	Bowel accident	0	0	0	0/130	0%	Very unlikely to occur
	Overall risk associated with scTS	2	7	6	15/130	11.53%	Unlikely to occur
Risks associated with AB-LT	Skin irritation from a harness	3	4	4	11/130	8.46%	Very unlikely to occur
	Skin irritation from trainers’ manual cues	0	1	2	3/130	2.31%	Very unlikely to occur
	Joint sprain	0	0	0	0/130	0%	Very unlikely to occur
	Bone fracture	0	0	0	0/130	0%	Very unlikely to occur
	Muscle soreness	0	0	0	0/130	0%	Very unlikely to occur
	Fall	0	0	0	0/130	0%	Very unlikely to occur
	Overall risk associated with AB-LT	3	5	6	14/130	10.77%	Unlikely to occur
	Other †	1	1	0	2/130	1.54%	Very unlikely to occur

Abbreviations: scTS—spinal cord Transcutaneous stimulation, AB-LT—Activity-based locomotor training, BP—blood pressure, HR—heart rate; †—P1 fell asleep, P14 felt cold with no change in body temperature; Data is reported as individual numbers of adverse occurrences for participants’ training sessions.

## Data Availability

All data collected during this study related to hemodynamic responses, incidence of pain, skin redness, autonomic dysreflexia, and other safety-related outcomes, as well as trunk kinematics and center of pressure displacement are openly available in Open Data Commons for Spinal Cord Injury (ODC-SCI.org) at DOI: 10.34945/F55P5X (ODC-SCI:1408). Raw participant demographic data are not publicly available due to privacy regulations. However, processed data used to generate the figures and tables in this manuscript are fully accessible at ODC-SCI. The Human Locomotion Research Center maintains an IRB-approved Volunteer Database (IRB #06.0647: Development of the KSCIRC Translational Research Database for Potential Research Volunteers), which allows members of the public to register as potential participants via https://victoryoverparalysis.org/participate-in-research/ (accessed on 13 June 2025). Access to use this database for research recruitment requires institutional IRB approval and is not publicly granted.

## Supplementary Materials

The following supporting information can be downloaded at: https://www.mdpi.com/article/10.3390/children12070817/s1, Table S1: EMG activity of trunk muscle in P1.
