# Supplementary material for: Cumulative Transcutaneous Spinal Stimulation with Locomotor Training Safely Improves Trunk Control in Children with Spinal Cord Injury: Pilot Study
Source: Children (Basel). 2025 Jun 21;12(7):817. doi: 10.3390/children12070817 (PMC12293838; doi:10.3390/children12070817)
Supplement: Supplementary file 1 [file children-12-00817-s001.zip › children-3635817-supplementary.pdf]

Supplementary Table 1. EMG activity of trunk muscle in P1

The root-mean-square of EMG, mV	Pre-post intervention	Quiet Sitting			Right Reach			Left Reach			Anterior Reach			Posterior Reach			One Arm Raise		
		**Mean**	**SD**	**N**	**Mean**	**SD**	**N**	**Mean**	**SD**	**N**	**Mean**	**SD**	**N**	**Mean**	**SD**	**N**	**Mean**	**SD**	**N**
**Right Oblique**	pre	10.1	0.4	3	11.1	1.3	3	13.6	0.8	3	11.7	2.2	3	8.1	0.2	3	6.1	0.2	3
	post-39	9.4	1.7	3	8.6	0.4	3	20.3	8.0	3	9.8	0.9	3	23.5	7.7	3	9.4	2.1	4
**Left Oblique**	pre	9.7	1.9	3	30.2	20.8	3	34.9	41.4	3	12.3	1.5	3	26.9	13.3	3	7.1	1.0	3
	post-39	5.9	0.7	3	19.4	8.0	3	7.5	0.6	3	8.7	0.8	3	10.1	1.6	3	7.2	1.2	4
**Right Rectus Abdominis**	pre	10.8	1.3	3	57.5	76.9	3	11.6	1.0	3	12.2	1.5	3	8.6	0.5	3	11.7	3.8	3
	post-39	9.2	2.3	3	11.4	3.9	3	9.4	3.7	3	8.1	0.3	3	11.3	2.8	3	8	1.6	4
**Left Rectus Abdominis**	pre	8.3	1.9	3	8.8	0.5	3	8	0.5	3	8.0	0.7	3	11.1	4.4	3	7.3	1.9	3
	post-39	5.2	1.1	3	7.2	1.3	3	8.2	3.5	3	6.4	1.2	3	6.3	0.5	3	6.3	1.3	4
**Right Erector spine (L5)**	pre	8.3	1.5	3	8.7	0.1	3	7.4	0.7	3	8.6	1.8	3	6.3	0.2	3	5.0	0.7	3
	post-39	6.0	1.2	3	5.4	0.3	3	9.3	6.0	3	6.2	0.2	3	6.5	0.1	3	5.6	1.2	4
**Left Erector spine (L5)**	pre	17.8	1.7	3	18.5	1.3	3	10.8	1.1	3	16.3	1.5	3	10.3	1.4	3	10.3	1.4	3
	post-39	11.2	2.2	3	15.3	1.0	3	7.1	0.9	3	15.0	1.4	3	14.3	12.7	3	12.6	3.2	4
**Right Erector spine (Th10)**	pre	50.3	8.8	3	40.4	1.4	3	47.2	3.2	3	50.1	10.2	3	40.9	6.2	3	19.0	2.5	3
	post-39	71.3	8.6	3	46.6	10.8	3	74.9	13.5	3	103.1	6.0	3	41.2	1.1	3	36.5	14.9	4
**Left Erector spine (Th10)**	pre	103.8	25.6	3	98.1	8.8	3	62.3	4.9	3	94.6	17.4	3	58.6	3.9	3	45.4	7.3	3
	post-39	185.9	38.2	3	239.3	9.5	3	82.2	15.6	3	245.1	19.6	3	83.5	5.4	3	207.7	40.4	4
**Right Upper Trapezius**	pre	20.7	2.3	3	32.7	7.6	3	197.8	77.3	3	24.8	12.2	3	170.1	104.7	3	428.8	40.7	3
	post-39	16.6	5.1	3	83.4	22.3	3	403.2	191.7	3	134.5	136.4	3	269.3	10.8	3	525.5	159.3	4
**Left Upper Trapezius**	pre	44.3	14.5	3	287.5	86.7	3	168.1	53.4	3	23.9	4.1	3	452.4	107.2	3	230.0	142.1	3
	post-39	23.5	12.5	3	187.6	30.3	3	146.2	86.5	3	120.9	63.0	3	79.8	9.3	3	118.1	84.1	4

Supplementary Table 2. EMG activity of trunk muscle in P14

**The root-mean-square of EMG, mV**	**Pre-post intervention**	**Quiet Sitting**			**Right Reach**			**Left Reach**			**Anterior Reach**			**Posterior Reach**			**One Arm Raise**		
		**Mean**	**SD**	**N**	**Mean**	**SD**	**N**	**Mean**	**SD**	**N**	**Mean**	**SD**	**N**	**Mean**	**SD**	**N**	**Mean**	**SD**	**N**
**Right Oblique**	pre	9.4	0.5	3	9.9	0.9	3	12.4	2.4	3	8.7	0.9	3	10.2	0.9	3	7.3	0.6	3
	post-39	9.9	0.8	3	12.7	1.4	3	14.5	1.4	3	10.8	0.4	3	9.2	0.8	3	16.9	1.8	3
**Left Oblique**	pre	21.1	0.3	3	24.2	1	3	14.6	2.5	3	18.7	3.5	3	15.0	1.8	3	14.1	5.0	3
	post-39	10.3	0.1	3	15.6	2.2	3	11.7	0.4	3	10.8	0.9	3	8.1	0.3	3	7.0	1.4	3
**Right Rectus Abdominis**	pre	8.0	1.5	3	8.6	0.6	3	7.5	1.3	3	7.0	0.7	3	7.7	0.2	3	6.4	0.1	3
	post-39	11.6	0.6	3	10.5	1.7	3	6.9	0.1	3	10.1	2.2	3	7.1	0.3	3	7.7	0.9	3
**Left Rectus Abdominis**	pre	6.3	0.0	3	8.1	0.9	3	7.0	1.9	3	6.8	0.6	3	10.0	1.9	3	5.7	0.7	3
	post-39	7.6	0.5	3	8.7	2.6	3	7.1	0.8	3	6.6	0.8	3	5.1	0.3	3	5.7	1.2	3
**Right Erector spine (L5)**	pre	32.3	3.8	3	33.3	8.5	3	32.0	6.6	3	29.8	3.9	3	23.1	2.3	3	7.8	1.6	3
	post-39	11.8	1.1	3	10.1	1.4	3	12.4	0.3	3	14.0	0.8	3	8.7	0.2	3	15.3	1.6	3
**Left Erector spine (L5)**	pre	12.2	0.7	3	12.8	0.6	3	8.9	2.0	3	10.9	2.3	3	7.6	0.8	3	7.4	1.8	3
	post-39	9.1	0.5	3	11.3	1.3	3	6.5	0.1	3	9.1	1.3	3	5.0	0.3	3	6.0	0.7	3
**Right Erector spine (Th10)**	pre	248.7	29.5	3	189.7	43.2	3	227.6	60.2	3	298.3	43.2	3	157.5	27.9	3	25.3	6.8	3
	post-39	148.0	7.0	3	80.4	8.0	3	114.6	5.0	3	171.6	19.4	3	62.0	10.1	3	94.3	5.5	3
**Left Erector spine (Th10)**	pre	116.7	13.1	3	114.6	25.8	3	68.2	37.4	3	113.7	30.7	3	57.2	14.6	3	69.6	35.2	3
	post-39	57.5	1.2	3	73.1	10.3	3	24.2	2.7	3	65.6	10.1	3	12.7	1.9	3	44.1	13.7	3
**Right Upper Trapezius**	pre	163.4	31.0	3	148.2	63.5	3	358.4	79.4	3	140.9	36.1	3	153.2	16.4	3	509.7	166.6	3
	post-39	35.4	8.0	3	223.9	143.6	3	405.1	122.3	3	156.3	86.1	3	186.1	39.8	3	619.2	105.6	3
**Left Upper Trapezius**	pre	106.1	18.0	3	184.6	84.0	3	161.6	95.9	3	132.8	65.6	3	98.1	49.6	3	130.5	108.7	3
	post-39	85.2	59.1	3	541.2	79.5	3	231.1	21.8	3	238.9	94.4	3	130.2	15.1	3	315.9	102.3	3

Supplementary Table 3. EMG activity of trunk muscle in P23

The root-mean-square of EMG, mV	Before-after COVID-19	Pre-post intervention	Timed Siting			Up-right sitting		
			Mean	SD	N	Mean	SD	N
**Right Oblique**	*bef. Cov-19*	pre	6.1	1.2	4			
	aft. Cov-19	pre	5.2	0.4	4	4.6	0.1	4
	aft. Cov-19	post-40	5.3	0.3	2	5.9	1.0	3
**Left Oblique**	*bef. Cov-19*	pre	5.6	0.6	4			
	aft. Cov-19	pre	6.5	0.8	4	5.5	0.3	4
	aft. Cov-19	post-40	5.1	0.1	2	5.5	0.3	3
**Right Rectus Abdominis**	*bef. Cov-19*	pre	7.4	3.9	4			
	aft. Cov-19	pre	7.0	0.3	4	6.6	0.4	4
	aft. Cov-19	post-40	6.4	0.2	2	7.0	0.1	3
**Left Rectus Abdominis**	*bef. Cov-19*	pre	20.3	28.5	4			
	aft. Cov-19	pre	6.3	0.4	4	6.2	0.6	4
	aft. Cov-19	post-40	7.9	0.2	2	9.3	0.1	3
**Right Erector spine (L5)**	*bef. Cov-19*	pre	5.3	0.3	4			
	aft. Cov-19	pre	5.5	0.4	4	5.2	0.2	4
	aft. Cov-19	post-40	5.6	0.2	2	6.1	0.5	3
**Left Erector spine (L5)**	*bef. Cov-19*	pre	5.2	0.4	4			
	aft. Cov-19	pre	5.0	0.1	4	4.8	0.1	4
	aft. Cov-19	post-40	3.0	0.0	2	2.9	0.2	3
**Right Erector spine (Th10)**	*bef. Cov-19*	pre	11.9	1.3	4			
	aft. Cov-19	pre	13.8	0.5	4	12.5	0.8	4
	aft. Cov-19	post-40	11.2	0.1	2	12.3	1.0	3
**Left Erector spine (Th10)**	*bef. Cov-19*	pre	12.2	1.2	4			
	aft. Cov-19	pre	15.2	3.3	4	13.5	2.3	4
	aft. Cov-19	post-40	15.2	0.0	2	15.9	2.2	3
**Right Upper Trapezius**	*bef. Cov-19*	pre	233.2	137.0	4			
	aft. Cov-19	pre	45.1	13.5	4	79.4	41.2	4
	aft. Cov-19	post-40	63.1	24.6	2	75.3	32.1	3
**Left Upper Trapezius**	*bef. Cov-19*	pre	189.6	100.4	4			
	aft. Cov-19	pre	57.7	44.0	4	63.1	54.3	4
	aft. Cov-19	post-40	28.8	6.7	2	102.7	39.1	3
